# Eugenesia y racismo científico en la investigación en torno a la nutrición y la alimentación en México

**DOI:** 10.1590/S0104-59702025000100016

**Published:** 2025-04-07

**Authors:** Ana María Carrillo

**Affiliations:** i Profesora titular, Departamento de Salud Pública/Facultad de Medicina/Universidad Nacional Autónoma de México. Ciudad de México – México, orcid.org/0000-0002-7981-2065, farga@unam.mx

**Figure:**
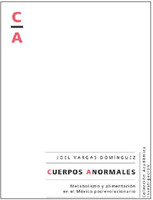
VARGAS DOMÍNGUEZ, Joel. *Cuerpos anormales: metabolismo y alimentación en el México posrevolucionario*. Ciudad de México: Universidad Autónoma Metropolitana/Unidad Cuajimalpa, 2023. 427p.

Este libro – que acaba de recibir el Premio Amilcar Cabral de ESOCITE 2024 – es resultado de la labor de investigación de un químico, historiador de la alimentación y filósofo de la ciencia, quien examina desde esos campos cómo se fueron construyendo redes internacionales que dieron sentido, orientaron y produjeron la investigación en torno a la nutrición y la alimentación en México, entre el fin de la Revolución y los años 1940.

Joel Vargas Domínguez (2023) describe cómo, a partir de estudios realizados en laboratorios de EEUU con hombres blancos, fueron delimitados los estándares normales metabólicos, y que, luego, investigadores estadounidenses decidieron estudiar a poblaciones diferentes, con la intención de mostrar que su metabolismo se salía de la norma; que sus cuerpos eran anormales.

El autor estudia tres proyectos que la Carnegie Institution of Washington realizó en Yucatán, con apoyo de científicos mexicanos, en 1927, 1929 y 1931 – el cual duró hasta 1937 –, todos los cuales tuvieron a la población blanca estadounidense como grupo de control. Explica que, con ellos, los investigadores estadounidenses intentaron usar el metabolismo como un parámetro claro, científico, en la delimitación racial; mostrar el efecto de los trópicos en la medición metabólica, así como delimitar lo normal de lo anormal del metabolismo a partir de las diferencias en la alimentación.

El estado nutricional de los mayas, de acuerdo con algunas clasificaciones, era satisfactorio; pero se les llamó – narra el autor – a seguir una dieta “racional”, a pesar de que ésta estuviera en contra de su consumo tradicional. Los cambios en la alimentación – asegura Joel Vargas – pueden considerarse eugenesia, y – como dice Gustavo Vallejo y coincide el autor – en ningún caso se trata de una eugenesia suave, sino de un poder ejercido sobre las poblaciones para controlarlas.

Vargas Domínguez muestra que las voces de las personas investigadas fueron silenciadas de los reportes científicos; por ejemplo, los investigadores decían creer más en la edad que para ellos aparentaban los individuos, que en la que los mismos individuos decían tener, o determinaban la adscripción racial por las características fenotípicas y no por la autoidentificación de los entrevistados como indígenas. Eliminaban aquello que no convenía a la investigación. Ya en su época estos estudios fueron criticados porque las conclusiones no se seguían de los datos experimentales, y es que detrás de las empresas de investigación científica había intereses coloniales, y en ellas intervenían ideas racistas, étnicas y de clase.

El libro reconstruye cómo se llegó a sostener la idea – luego generalizada – de que una correcta alimentación era la que incluía alimentos de origen animal; aclara que detrás de esta política estaban los ganaderos, que habían patrocinado las investigaciones, y que después de establecer que esa era la dieta normal, con criterios supuestamente científicos, los investigadores afirmaron que las dietas de los indígenas y de los pobres eran malas, y podían explicar la supuesta degeneración racial de esas comunidades o grupos de población.

Otros estudios de metabolismo indígena – también analizados por el autor – fueron realizados en la década de 1930 en un grupo de más de cien otomíes habitantes del Valle de Mezquital, en el estado de Hidalgo, una de las poblaciones más pobres del país, a la que se calificó de “primitiva”. Se les estudió antropométrica y fisiológicamente como parte de un “grupo étnico”, con lo cual los investigadores pretendían distanciarse del concepto de “raza”. De nuevo, hubo redes de investigadores nacionales e internacionales, en este caso franceses, que afirmaron lo que la Carnegie había dicho para los mayas: el metabolismo basal de los indígenas era diferente a lo que se esperaba de un metabolismo considerado normal. La idea era que el indígena podía ser estudiado y medido para conocer su supuesto grado de degeneración y el Estado debía cuidarlo y mejorarlo. Se llegó – arguye el autor – con ideas preexistentes de que su alimentación era inadecuada, y hubo intervenciones alimentarias enfocadas en el incremento de consumo de proteínas animales con especial atención a la leche.

Después, con apoyo de la Fundación Rockefeller, investigadores mexicanos realizaron estudios con poblaciones mestizas, que son las últimas analizadas en el libro. Hubo un proyecto de nutrición para los pobres de la Ciudad de México, que fue la creación de comedores públicos con fines de experimentación sobre la población, a través de los cuales se realizaron estudios de la alimentación colectiva, y que buscaba también educar a los beneficiarios de esos programas y mejorar su cuerpo para integrarlos a la nación. Se pensaba que la alimentación podía incidir en las enfermedades pero también ayudaba al control de éstas, por ejemplo en el caso de la diabetes.

Cronológicamente, el libro termina en 1943, con la creación del Instituto Nacional de Nutriología, primer instituto de nutrición mexicano, del que Joel Vargas Domínguez (2019) ha sido el principal estudioso. Sin embargo, el autor incluye también reflexiones sobre la época actual, en que el Estado ha establecido políticas públicas de alimentación que controlan el acceso a los alimentos ultra procesados, al tiempo que responsabiliza a los consumidores de su sobrepeso u obesidad y, por lo tanto, de las enfermedades metabólicas.

El libro presenta cuadros y fotografías, las cuales son analizadas en el texto. La investigación está basada en fuentes primarias localizadas en archivos mexicanos y estadounidenses, además del archivo de la Organización Mundial de la Salud, así como en fuentes secundarias relativas a la historia de la construcción de la normalidad y anormalidad metabólica, la eugenesia y la herencia biológica, a las que el autor retoma, y con las que también debate.

Estudiando ejemplos locales, pero a partir de intercambios internacionales, muestra como la ciencia, que siempre se ha pretendido científica, ha empleado criterios raciales, étnicos o de clase, para definir como inferiores a poblaciones diferentes, entre ellas las de los indígenas y los pobres, y que los estudios de fisiología y alimentación, en particular, contribuyen a reconstruir la historia de la eugenesia y del racismo en el periodo de entreguerras en México. Pienso que esta obra resultará de gran utilidad para quienes realizan estudios de eugenesia, y de historia de la salud pública, así como para quienes se ocupan de la emergencia de las ciencias de la alimentación.
